# Attenuated Combined Action of Cyclosporine A and Hyperlipidemia on Atherogenesis in Rabbits by Thymoquinone

**DOI:** 10.1093/ecam/nep225

**Published:** 2011-05-03

**Authors:** Ahmed Ragheb, Ahmed Attia, Fawzy Elbarbry, Kailash Prasad, Ahmed Shoker

**Affiliations:** ^1^Department of Medicine, Royal University Hospital, University of Saskatchewan, Saskatoon, SK, Canada; ^2^School of Pharmacy, Pacific University, Hillsboro, OR, USA; ^3^Department of Physiology, College of Medicine, University of Saskatchewan, Saskatoon, SK, Canada

## Abstract

This descriptive study investigates in a rabbit model of atherosclerosis (i) the extent of atherogenesis induced by cyclosporine A (CsA) or hyperlipidemia alone or in combination and (ii) whether thymoquinone (TQ), a known herbal antioxidant, offers protection against these effects. New Zealand White female rabbits were assigned to five groups of six animals each: Group I, control; Group II, CsA [25 mg kg^−1^ day^−1^ orally (PO)]; Group III, 1% cholesterol; Group IV, 1% cholesterol + CsA (25 mg kg^−1^ day^−1^ PO); and Group V, 1% cholesterol + CsA (25 mg kg^−1^ day^−1^ PO) + TQ (10 mg kg^−1^ day^−1^ PO). Lipids and oxidative stress parameters [Malondialdehyde (MDA) and protein carbonyl] and aortic atherosclerosis were compared. CsA alone did not show a significant effect on either serum lipids and did not induce atherosclerosis. High-cholesterol diet induced atherosclerosis (45 ± 11% of the intimal surface of aorta was covered with atherosclerotic plaques). CsA and high-cholesterol diet increased atherosclerosis severity as measured from intimal and media lesions, but did not affect the extent of atherosclerosis. TQ decreased aortic MDA by 83%. It was also associated with reduced aortic atherosclerosis extend by 52% compared with Group IV. We concluded that (i) CsA aggravates hyperlipidemia-induced atherosclerosis and (ii) TQ attenuates the oxidative stress and atherogenesis induced by the combined effect of CsA and hyperlipidemia.

## 1. Introduction

Patients receiving calcineurin inhibitors such as cyclosporine A (CsA) including transplant patients and those with immune-mediated kidney diseases are usually hyperlipemic. Both of hyperlipidemia and CsA contribute to the increased cardiovascular burden in humans and animal models [1–3]. Therefore, the relative contribution of CsA versus hyperlipidemia alone or in combination to the cardiovascular disease burden is difficult to dissect in humans. Adding to this complexity is the fact that both hyperlipidemia and CsA causes tissue injury by increasing the oxidative stress. Furthermore, the effect of CsA on atherosclerosis is complex. On one hand, CsA inhibits T-cell function and thereby recruitment of inflammatory cells needed to initiate atherosclerosis [4], which depends on T-cell activation. On the other hand, CsA has been demonstrated to synergistically exacerbate the vasoconstrictive effects of oxidized low-density lipoprotein [5, 6] and thus aggravates atherosclerosis.

To determine the relative contribution of CsA and hyperlipidemia to induce atherosclerosis, we reasoned that high cholesterol-fed rabbits are a good model to study. First, rabbits have been used reliably to induce an atheroma model with intact endothelium, which resembles human atherosclerosis [7, 8]. Secondly, oxidative stress has been linked to tissue injury by hyperlipidemia [9, 10]. And thirdly, we have demonstrated that thymoquinone (TQ), which is the bioactive constituent of the volatile oil of *Nigella sativa* seeds, protects against hyperlipidemia-induced atherosclerosis in rabbits. This effect is likely due to attenuation of oxidative stress [11, 12].

We therefore, wished to test the hypothesis that CsA aggravates hyperlipidemia-induced atherosclerosis by synergistically enhancing the reactive oxygen species (ROS) activity and that TQ ameliorates these effects. In this study, we aimed to describe these effects.

## 2. Methods

### 2.1. Drugs

The drugs used were CsA A (CsA):Neoral oral solution (100 mg ml^−1^) (Novartis Pharmaceuticals Canada Inc., Dorval, QC, Canada) and TQ: 2-isopropyl-5-methyl-1,4-benzoquinone (Sigma-Aldrich, St. Louis, MO, USA) dissolved in pure corn oil (Sigma-Aldrich, St Louis, MO 63103, USA) to give a concentration of 75 mg ml^−1^.

### 2.2. Animals and Experimental Design

Thirty female New Zealand White rabbits, weighing 1.8–2 kg and aged 6–8 weeks, were assigned to five groups of six animals each (Table 1). After 1 week of adaptation on regular diet. The rabbits were assigned to the following groups: Group I, control; Group II, CsA (25 mg kg^−1^ day^−1^ per 8 weeks PO); Group III, 1% cholesterol; Group IV, 1% cholesterol + CsA (25 mg kg^−1^ day^−1^ per 8 weeks PO); and Group V, 1% cholesterol + CsA (25 mg kg^−1^ day^−1^ 8 weeks PO) + TQ (10 mg kg^−1^ day^−1^ per 8 weeks PO). The diet was prepared by Purina (St Louis, MO, USA) and did not contain any antioxidants. Drugs were given orally through nasogastric tubes (water was given *ad libitum*). The rabbits were housed in individual cages at room temperature of 22–24°C and a relative humidity of 40–60% under a 12-h light/12-h dark cycle. The experimental protocols were approved by the Ethics Committee of the University of Saskatchewan and the animal care was performed according to the approved standards for Laboratory Animal Care. Following 18 h of fasting, blood samples (from the ear marginal artery) were collected before (0 time) and after 4 and 8 weeks on the respective diets for measurement of total cholesterol (TC), triglycerides (TG), low density lipoprotein-cholesterol (LDL-C), high density lipoprotein-cholesterol (HDL-C), TC/HDL-C and serum malondialdehyde (MDA). Serum protein carbonyl was measured at week 8 only. At the end of the protocol (8 weeks) rabbits were anesthetized with ethanol, pentobarbital sodium (50 mg kg^−1^ IV) (Bimeda-MTC Animal Health Inc., Cambridge, ON, Canada) in the marginal ear vein and aortas were removed for the assessment of aortic MDA and protein carbonyl and atherosclerotic changes.

### 2.3. Serum Lipids

TC, TG and HDL-C were measured on an automated Beckman Synchron LX20 Clinical System Analyzer. LDL-C was calculated. Cholesterol risk ratio was estimated using the TC/HDL-C ratio.

### 2.4. Preparation of the Aortic Tissue for Measurement of MDA and Protein Carbonyl

The aortic rings were cut at the roots of the aortic arches and kept in buffered 10% formalin for histological examination using our previously described methods [9, 13].

Aortas between the origin and bifurcation to iliac arteries were removed, cleaned of gross adventitial tissue and cut longitudinally into two halves. One half was used for the assessment of atherosclerotic changes, and the other half was kept on ice for preparation of the supernatants, by a previously described method [14], for measurement of the aortic tissue MDA and protein carbonyl.

### 2.5. Serum and Aortic MDA (Thiobarbituric Acid-Reactive Substances)

MDA levels in the aortic tissue supernatant and serum were measured as thiobarbituric acid-reactive substances (TBARs) by the previously described method [14, 15]. The MDA content of the tissue was expressed as nmol mg^−1^ proteins and that of serum as nmol ml^−1^.

### 2.6. Serum Protein Carbonyl

The protein carbonyl levels in the serum were measured using a Cayman Chemical Carbonyl Protein Assay Kit (Cayman Chemical Company, Ann Arbor, Michigan, USA). The absorbance was measured at a wavelength between 360 and 386 nm using an ELx 808TM Absorbance microplate reader (BioTek Instruments, Inc. Highland Park, Vermont, USA). The protein carbonyl content of the tissue was expressed as nmol mg^−1^ proteins and that of serum as nmol ml^−1^.

### 2.7. Tissue Protein Measurement

The protein content of was determined using a Modified Lowry Protein Assay Kit (Pierce Biotechnology, Rockford, IL, USA). The absorbance was measured at a wavelength of 750 nm using an ELx 808TM Absorbance microplate reader (BioTek Instruments, Inc., Highland Park, Vermont, USA).

### 2.8. Assessment of Atherosclerotic Changes in the Aorta

The six animals from each group were assessed for atherosclerotic changes using Herxheimer's solution containing Sudan IV for lipid staining [9, 16]. Photographs of the stained intimal surface of the aorta were taken with a digital camera. The total and atherosclerotic areas of the intimal surface of the aorta were measured by using image analysis software Scion Image for Windows (Scion Corporation, Frederick, MD USA). The extent of atherosclerosis was expressed as a percentage of total intimal surface area.

### 2.9. Quantification and Histological Assessment of Atherosclerotic Lesions in the Aortic Rings

All methods are well established at our laboratory [9]. The aortic rings specimens were cut across and embedded in paraffin. The histopathological findings were evaluated by a blinded pathologist.

H&E, Verhoeff's elastic stained sections were used for microscopic morphometric evaluation using the Scion software program. For these experiments, data have been reported as the mean intimal area, determined by six experimental measurements [17]. Video microscopy was used to outline the endothelium, internal elastic lamina (IEL) and external elastic lamina (EEL). Then, area measurements were calculated using an image software program (Scion, Frederick, MD). Intima area (between the endothelium and IEL) and media area (between the IEL and EEL) were measured for each aorta and averaged for each group.

The total cross-sectional intimal area was measured between the endothelial cell monolayer and the internal elastic lamina (IEL) and the total cross-sectional medial area was measured between the external elastic lamina (EEL) and the IEL as documented by previous methods [18]. The intimal and medial surface areas as well as the intima/media ratio were taken as measures of the severity of atherosclerosis [18].

### 2.10. Statistical Analysis

Results are expressed as mean ± SD. Repeated-measures analysis of variance was used for statistical analysis. A value of *P* < .05 was considered significant.

## 3. Results

### 3.1. Body Weight

There was a progressive increase in the body weight of all groups. No significant differences were observed between the groups throughout the study period (data not shown).

### 3.2. Serum Lipids

**Figure 1:**
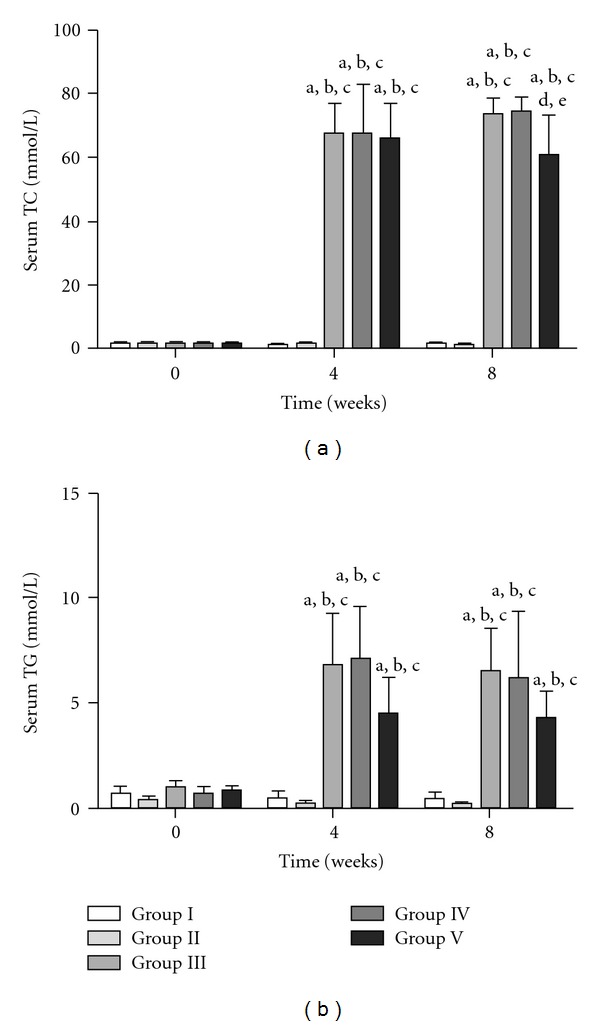
Sequential changes in serum TC and TG in the five groups. Results are expressed as mean ± SD. ^a^ *P* < .05, 0 time versus 4 and 8 weeks in the respective groups. ^b^ *P* < .05, Group I versus other groups. ^c^ *P* < .05, Group II versus Group III, IV or V. ^d^ *P* < .05, Group III versus Group IV or V. ^e^ *P* < .05, Group IV versus Group V.

The basal levels of serum TC in Groups I, II, III, IV and V were 1.38 ± 0.2, 1.35 ± 0.3, 1.26 ± 0.18, 1.35 ± 0.3 and 1.42 ± 0.4 mmol l^−1^, respectively. The basal levels were not significantly different among the groups. The sequential changes in serum TC in the five groups are shown in Figure 1. The serum levels of TC remained unchanged in Groups I and II while they were elevated in Groups III, IV and V as compared to their basal levels. The levels at week 8 were not different from those at week 4 in all groups. In Group V, TC was significantly lower than that for Groups III and IV at week 8.

The initial levels of serum TG in Groups I, II, III, IV and V were 0.79 ± 0.2, 0.94 ± 0.5, 1.02 ± 0.3, 0.74 ± 0.3 and 0.85 ± 0.2 mmol l^−1^, respectively. These initial levels were not significantly different among the groups. The sequential changes in serum TG in the five groups are shown in Figure 1. The serum levels of TG remained unchanged in Groups I and II while they were elevated in Groups III, IV and V as compared to their basal levels. No significant differences were found between the five groups at 4 and 8 weeks.

The basal levels of serum LDL-C in Groups I, II, III, IV and V were 0.4 ± 0.2, 0.49 ± 0.2, 0.35 ± 0.2, 0.43 ± 0.1 and 0.51 ± 0.4 mmol l^−1^, respectively. These levels were not significantly different from each other. The sequential changes in LDL-C in the five groups are shown in Figure 1. Serum levels of LDL-C remained unchanged in Groups I and II while they were elevated in Groups III, IV and V compared with their basal levels. No significant differences were found between the five groups at 4 and 8 weeks.

The initial levels of serum HDL-C in Groups I, II, III, IV and V were 0.62 ± 0.2, 0.51 ± 0.4, 0.44 ± 0.2, 0.58 ± 0.1 and 0.52 ± 0.1 mmol l^−1^, respectively. Again, these levels were not significantly different among the groups. The sequential changes in HDL-C in the five groups are shown in Figure 1. Serum levels of HDL-C remained unchanged in Groups I and II while they were elevated in Groups III, IV and V compared to their basal levels. In Group V, HDL-C was significantly lower than that in Groups III and IV at both 4 and 8 weeks.

**Figure 2:**
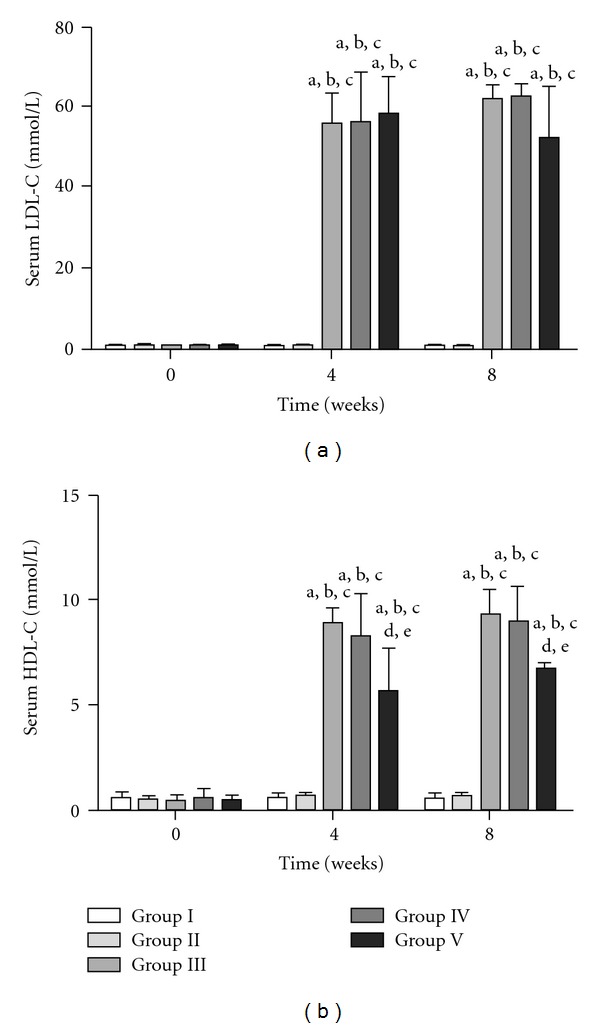
Sequential changes in serum LDL-C and HDL-C in the five groups. Results are expressed as mean ± SD. ^a^ *P* < .05, 0 time versus 4 and 8 weeks in the respective groups. ^b^ *P* < .05, Group I versus other groups. ^c^ *P* < .05, Group II versus Group III, IV or V. ^d^ *P* < .05, Group III versus Group IV or V. ^e^ *P* < .05, Group IV versus Group V.

The basal values of TC/HDL-C ratios in Groups I, II, III, IV and V were 2.31 ± 0.4, 2.64 ± 0.8, 3.04 ± 1, 2.35 ± 0.3 and 2.75 ± 0.5, respectively. The basal values were not significantly different among the groups. The sequential changes in these ratios in the five groups are shown in Figure 2. The ratios remained unchanged in Groups I and II and increased in Groups III, IV and V with respect to zero time. The risk ratio values were increased in Groups III, IV and V as compared to those in Groups I and II at 4 and 8 weeks. There were no significant differences between Groups III, IV and V at 8 weeks.

### 3.3. MDA Levels

#### 3.3.1. Serum MDA

The basal values of serum MDA in Groups I–V were 0.93 ± 0.4, 1.08 ± 0.5, 0.84 ± 0.3, 0.86 ± 0.2 and 0.79 ± 0.2 nmol l^−1^, respectively. The sequential changes in serum MDA in the five groups are shown in Figure 2. The values remained unchanged in Group I but increased in the other Groups at 4 and 8 weeks with respect to baseline values. The increases in MDA levels in Groups III and V were similar. MDA levels were the highest in Group IV at 4 and 8 weeks.

#### 3.3.2. Aortic Tissue MDA

The MDA contents of the aorta in the five groups are shown in Table 2. In Group 1 it was 0.25 ± 0.06 nmol mg^−1^ protein. In Groups II and III, the MDA levels were similar but lower than that in Group IV. Respectively MDA levels in Groups II, III and IV were 2.1-, 2.2- and 7.3-folds, respectively, higher as compared with Group I. The MDA level in Group V was 83% lower than that in Group III compared to Group IV, while it was similar to that in Group I.

### 3.4. Serum Protein Carbonyl Levels

The serum protein carbonyl levels in the five groups at 8 weeks are given in Table 2. Serum protein carbonyl level in Group I was 1.34 ± 0.25. The CsA and cholesterol diet increased the serum protein carbonyl levels by 5.75- and 3.92-fold, respectively. The levels in Group IV increased 11.33-fold as compared with Group I while the levels were 2- and 2.9-fold higher as compared with Groups II and III respectively. The serum protein carbonyl levels were 38.8% lower as compared to Group IV.

### 3.5. Histological Effects

#### 3.5.1. Extent of Atherosclerotic Plaque

**Figure 3:**
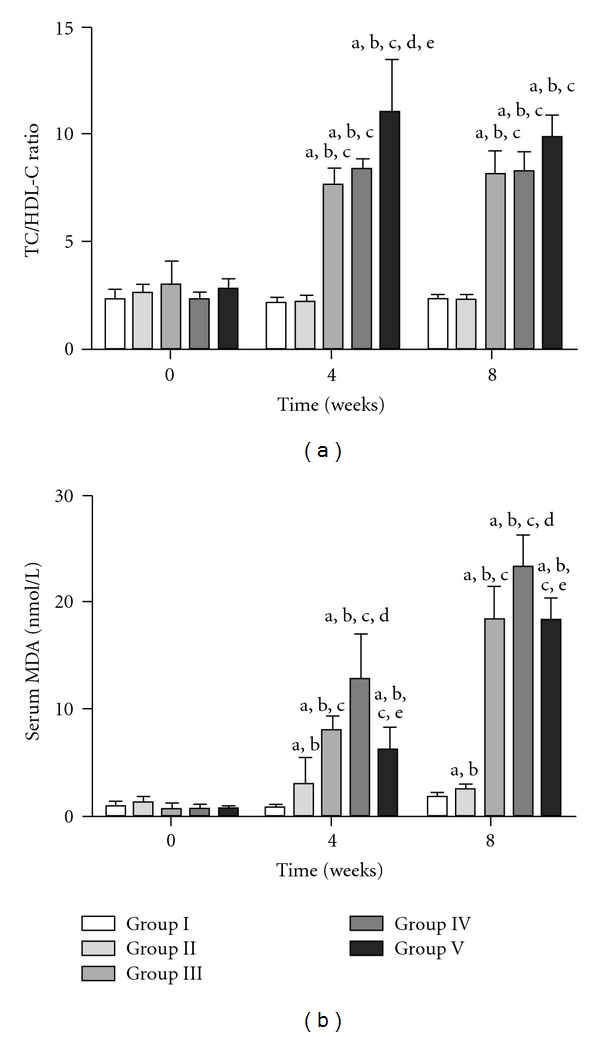
Sequential changes in TC/HDL-C ratio and serum MDA in the five groups. Results are expressed as mean ± SD. ^a^ *P* < .05, 0 time versus 4 and 8 weeks in the respective groups. ^b^ *P* < .05, Group I versus other groups. ^c^ *P* < .05, Group II versus Group III, IV or V. ^d^ *P* < .05, Group III versus Group IV or V. ^e^ *P* < .05, Group IV versus Group V.

Representative photographs of the atherosclerotic changes in the intimal surface of the aortas from the five groups are shown in Figure 3 and the results are summarized in Table 2. There were no atherosclerotic changes in the intimal surface of the aortas in Groups I and II. Significant areas of the intimal surfaces of Groups III (45 ± 11%), IV (38 ± 4%) and V (18 ± 8%) were covered with atherosclerotic plaques. TQ administration (Group V) reduced the development of cholesterol + CsA-induced atherosclerosis by 52%.

#### 3.5.2. Microscopic Changes in the Aortic Segments

**Figure 4:**
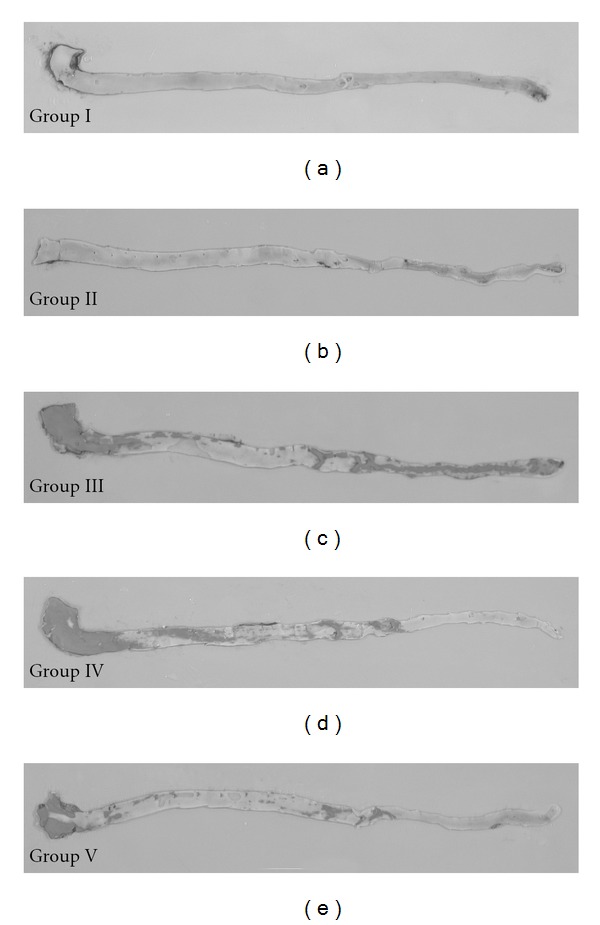
Representative photographs of the intimal surfaces of aortas from the five experimental groups showing Sudan IV-stained lipid deposits. The brick-red lipid deposits in Groups III, IV and V.

**Figure 5:**
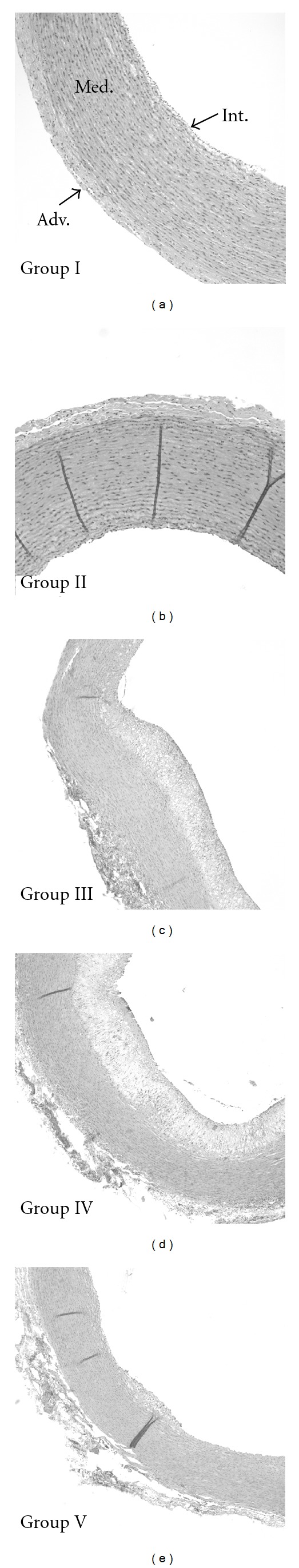
Representative photographs of the microscopic changes from the five groups stained with H&E stain. Note the normal arterial wall structure in Group I, the increased intimal thickness in Group III that involves a large portion of the aortic circumference and almost the whole circumference in Group IV. Also note the very small atheroma in Group V (magnification, 4x).

**Figure 6:**
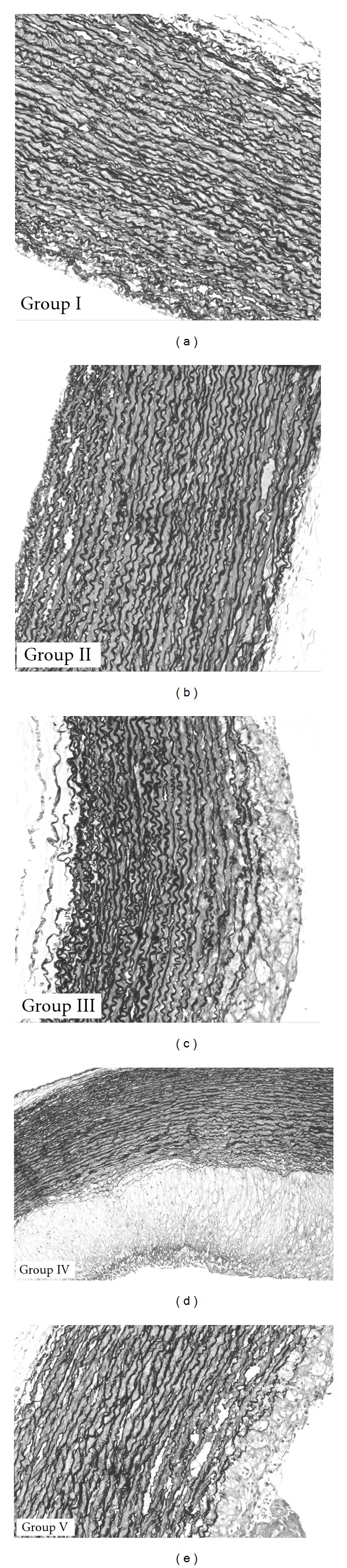
Representative photographs of the microscopic changes from the five groups stained with Verhoeff's elastic stain with magnification 10 and 20x. *Note:* (i) the normal arterial wall structure in Group I; (ii) the moderate to sever atherosclerosis in Group III that involves a large portion of the aortic circumference; (iii) severe atherosclerosis in Group IV and the smooth muscle cells proliferation in the intima; (iv) also note the very small atheroma in Group V and the absence of smooth muscle cells proliferation in the intima. Mild ≤ 30%, moderate = 30–60%, severe ≥ 60%.

Histological examination of all animals in each group was first performed. Representative photographs of the histological sections of the aorta stained with the H&E stain and Verhoeff's elastic stain from the five groups were photographed and are shown in Figures 4, 5, and 6. In the control group (Group I), specimens showed normal arterial wall structure with no atherosclerotic changes. Administration of CsA alone in Group II had no effect on the atherosclerosis parameters tested. In Group III moderate (30–60%) to severe (≥60%) atherosclerotic lesions was obvious. Administration of CsA with the high-cholesterol diet in Group IV disclosed severe diffuse atherosclerosis involving >85% of the arterial wall and, in addition, smooth muscle cells proliferation in the intima. Co-administration of TQ to CsA and the high-cholesterol diet attenuated the vascular injury to only minimal focal areas of atherosclerosis involving <20% of the arterial wall. In addition there was no evidence of smooth muscle cells proliferation.

#### 3.5.3. Changes in the Intimal and Medial Thickness in the Aortic Segments

Three parameters were compared; the intimal and medial thickness of the aortic specimens as well as the intimal/medial ratio. The results are summarized in Table 2. The intimal and medial cross-sectional areas and the values of intima/media ratio in Group I were 7.6 ± 1.5 ×10^3^ *μ*m^2^, 52.3 ± 9.7 × 10^3^ *μ*m^2^ and 0.15 ± 0.01, respectively. CsA alone did not exert any significant effect. In Group III, the intimal cross-sectional area and the intima/media ratio values were increased 4.3- and 3.5-fold, respectively, compared to those in Group I. Administration of CsA to the high-cholesterol diet, in Group IV, significantly increased the intimal and medial cross-sectional areas and the intima/media ratio 1.8, 1.4 and 1.3 times, respectively, compared to that in Group III. Adding TQ in Group V to both CsA- and cholesterol-rich diet decreased the intimal cross-sectional area and the intima/media ratio values by 73 and 72%, respectively, compared to that in Group III.

#### 3.5.4. Changes in the IEL and EEL in the Aortic Segments with Verhoeff's Elastic Stain

Administration of CsA only in Group II had no effect. The high-cholesterol diet administration in Group III produced diffuse damage to the IEL at the sites of the atherosclerotic changes. Combining CsA to the high-cholesterol diet disclosed diffuse damage to the IEL. Co-administration of TQ to the CsA and the high-cholesterol diet reduced the damage to minimal focal areas of disruption in the IEL.

## 4. Discussion

This study highlights certain points for discussion; first, there was no significant difference in the body weight gain among the studied groups (data not shown) indicating that the various interventions did not affect the body weight.

Second, our results corroborate similar effects of the high-cholesterol diet on the lipid profile as reported in previous studies [19, 20].

This study demonstrates some of the biological effects of clinical relevance, on CsA toxicity. First, CsA at the dose used did not affect lipid levels and thus its effect on the biochemical changes and tissue damage as reported in Group II are not compounded by changes in serum lipid levels. Among these biological effects this study corroborates the increased oxidative state after CsA administration. This was well manifested by an increase in both serum MDA and protein carbonyl levels by 1.4- and 5.8-fold, respectively, when compared to the control group. In addition, CsA increased the aortic MDA level 1.9-fold, but this level of enhanced ROS activity was not high enough to induce atherosclerosis. When CsA action was compared to that induced by the high cholesterol diet the results demonstrated a much higher level of oxidative stress induced by the high cholesterol diet. The combination of CsA and high cholesterol diet exerted a synergistic effect on oxidative state as manifested by an increase in serum MDA and protein carbonyl as well as the aortic tissue MDA levels by 9.2-, 2- and 3.9-fold, respectively, when compared to that in Group II (CsA group) or when compared to the high cholesterol group (1.3-, 2.9- and 2.3-fold, resp.).

Thus, the results proof the synergistic effect of CsA and high cholesterol diet on increasing oxidative stress. Other studies corroborated the deleterious combination of hyperlipidemia and CsA on atherosclerosis [21].

We have recently demonstrated that TQ inhibits the formation of atherosclerotic plaque in this model by 50% [12]. In this current study, the addition of CsA further enhanced the protective effect of TQ in the same model by 80%. Taken together, the results raise the intriguing appeal that while TQ may abolish some of the deleterious effects of combined administration of CsA and hyperlipidemia, TQ spares also or even unmasks some of the desirable effects of CsA such as the inhibition of atherosclerosis. The anti-atherogenic effect of CsA when used in combination with TQ is both intriguing and may have significant clinical relevance. First, there is a plethora of evidence indicating a role for the immune system in the development of vascular disease. Thus, suppression of cell-mediated immunity by CsA induced inhibition of T-lymphocytes [4, 22–24].

In this study, we used other parameters to assess the development of atherosclerosis; the intimal and medial cross-sectional areas and the intima/media ratio. These parameters were not tested in previous studies [4, 7]. A main finding of this work was the beneficial effect of TQ on atherogenesis parameters. First, it inhibited markers of oxidative stress which included a decrease in the aortic tissue MDA content by 84%. Another striking finding of our work is the effect of TQ in this model. TQ in Group V reduced the extent of atherosclerosis by half and induced *∼*75% reductions in the intimal cross-sectional area and the intima/media ratio as compared to CsA and the high cholesterol diet in Group IV. Thus, these findings establish the useful effect of TQ to attenuate atherogenesis induced by hyperlipidemia which is aggravated by CsA.

Our results differ from those obtained by Drew and Tipping who studied the effect of CsA on a rabbit model similar to ours [6]. They used 1% cholesterol diet for 4 weeks to induce atherosclerosis in New Zealand white rabbits. CsA was given at a dose of 16 mg kg^−1^ of body weight IM every second day for 4 weeks. In their study they reported that CsA reduced the atherosclerotic plaques development in the aortic arch, the thoracic and abdominal aorta by 55, 68 and 86%, respectively. It also reduced the cross-sectional plaque area in the aortic arch by 55%. They attributed these effects to the anti-inflammatory effects of CsA through the prevention of T-cells recruitment and reduction of foam cells accumulation in the atherosclerotic plaques. It is possible that differences between our results and theirs might be explained by the lower dose and the shorter duration they used. This explanation is corroborated with the results of Roselaar and co-workers who described an atherogenic effect of a larger dose CsA in the same animal model [4]. This group induced atherosclerosis by 0.5% cholesterol diet and gave CsA at a higher dose (20 g kg^−1^ subcutaneously daily for the first week, and then twice weekly) for a longer duration (12 weeks). They reported that CsA increased the extent of atherosclerosis in the aortic arch and the thoracic aorta by 1.25- and 1.7-fold, respectively, as compared to the high-cholesterol diet alone. From what is said before we postulate that the effect of CsA on the extent of atherosclerotic plaques in cholesterol-fed animal models may depend on the duration and the CsA dose. When it is given in a low dose and for a short duration, CsA may exert an anti-atherogenic effect while, a further increase in the duration and dose induces atherogenesis.

**Figure 7:**
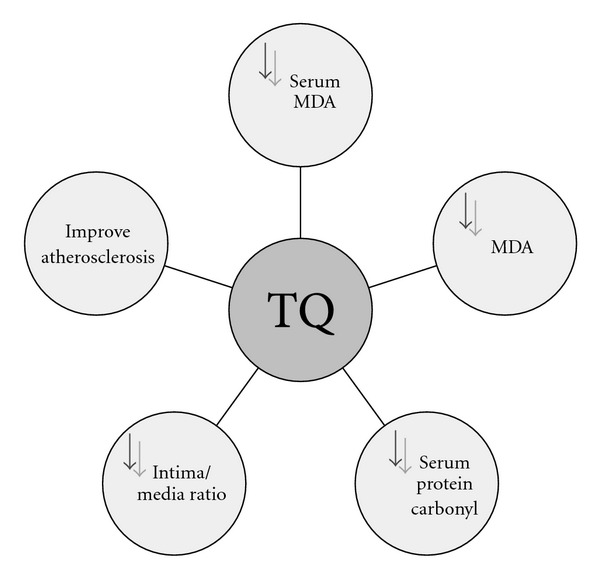
Hypothetical diagram to present the beneficial effect of thymoquinone.

Clinical relevance of these finding as shown in Figure 7 include the possible therapeutic use of TQ in hyperlipidemic patients on CsA therapy. Thus further controlled studies in humans are suggested.

### 4.1. Limitations

Caution should be exercised to interpret our results. We did not measure the oxidized LDL-C levels. Oxidation of LDL-C molecules has been recognized as a major atherogenic factor in post-transplant patients [25] and is considered a key factor in the biological processes that trigger and accelerate atherosclerosis [26]. Furthermore, we neither measured tissue CsA levels nor tested the effect of varying CsA dosing in our model. Currently experiments are performed to address these limitations and to delineate the underlying mechanisms of the beneficial effects observed in this descriptive study.

## 5. Conclusion

Enhanced oxidative stress is blamed for both hyperlipidemia and CsA. In reality, most patients on CsA are also hyperlipemic. Therefore, the contribution of CsA versus hyperlipidemia-induced damage is difficult to dissect. Similarly, in rabbits, hyperlipidemia has been reliably shown to cause tissue injury due to enhanced oxidative stress. Therefore, we raised the intriguing question to determine the relevance of CsA to vascular injury in the presence or absence of hyperlipidemia.

CsA administration alone induced oxidative stress but did not induce significant effect on the serum lipids or the development of atherosclerosis. However, the high-cholesterol diet induced a significant increase in parameters of oxidative stress as well as serum levels of TC, TG, LDL-C, HDL-C, the TC/HDL-C ratio. In addition, it markedly enhanced atherogenesis. Thirdly, the addition of CsA to high cholesterol diet led to a further amplification in the oxidative stress and, thus, severe atherogenesis. A main finding of this study is that TQ significantly attenuated aortic MDA levels and atherosclerosis in the presence of the deleterious combination of CsA and hyperlipidemia.

This study suggests TQ as a potential agent to protect against the deleterious effect of combined CsA and hyperlipidemia on atherosclerosis.

## Funding

Wyeth Canada and Novartis Canada for their unrestricted grants (fund numbers: Wyeth Canada: 402644/E.C, 2004-197 and Novartis Canada: 405710/E.C, 2005-130).

## Figures and Tables

**Table 1:** Experimental groups.

Group	Diet and drug regimen
I (*n* = 6)	Control; regular diet for 8 weeks
II (*n* = 6)	CsA; CsA [25 mg kg^−1^ day^−1^ orally (PO)]
III (*n* = 6)	Cholesterol; 1% cholesterol diet for 8 weeks
IV (*n* = 6)	Cholesterol + CsA; 1% cholesterol + CsA (25 mg kg^−1^ day^−1^ PO)
V (*n* = 6)	Cholesterol + CsA + TQ; 1% cholesterol + CsA (25 mg kg^−1^ day^−1^ PO) + TQ (10 mg kg^−1^ day^−1^ PO)

*n* = number.

**Table 2:** Aortic atherosclerotic changes and MDA and serum protein carbonyl levels in the five groups at 8 weeks.

	Group I	Group II	Group III	Group IV	Group V
	Control	CsA	Cholesterol	Cholesterol + CsA	Cholesterol + CsA + TQ
Atherosclerosis	0	0	45.08 ± 11.65*	38.25 ± 8.74^∗†^	18.41 ± 2.38^∗‡§^
(% of total intimal surface)					
Intimal area (×10^3^ *μ*m^2^)	7.58 ± 1.52	4.72 ± 0.752	32.38 ± 7.13*	58.48 ± 15.51^∗‡^	14.82 ± 1.87^∗‡§^
Medial area (×10^3^ *μ*m^2^)	52.31 ± 9.71	58.113 ± 8.89	64.56 ± 10.88	88.32 ± 10.08^∗‡^	82.42 ± 4.71^‡^
Intima/media ratio	0.15 ± 0.01	0.08 ± 0.02	0.52 ± 0.06*	0.65 ± 0.12^∗‡^	0.18 ± 0.03^∗‡§^
Aorta MDA (nmol mg^−1^)	0.25 ± 0.06	0.47 ± 0.26*	0.53 ± 0.32*	1.83 ± 0.68^∗†‡^	0.30 ± 0.03^†‡§^
Serum protein carbonyl content	1.34 ± 0.25	7.88 ± 1.88*	5.34 ± 1.36*	15.37 ± 1.29^∗†‡^	9.50 ± 1.74^∗†‡§^
(nmol ml^−1^)					

Results are expressed as mean ± SD. Intimal and medial surface areas are expressed as ×10^3^ *μ*m^2^.

*P < .05, Group I versus other groups.

^†^ *P* < .05, Group II versus Groups III, IV and V.

^‡^ *P* < .05, Group III versus Groups IV and V.

^§^ *P* < .05, Group IV versus Group V.
